# Effects of a blended learning approach on student outcomes in a graduate-level public health course

**DOI:** 10.1186/1472-6920-14-47

**Published:** 2014-03-11

**Authors:** Marc T Kiviniemi

**Affiliations:** 1 Department of Community Health and Health Behavior, University at Buffalo, 314 Kimball Tower, 3435 Main Street, Buffalo, NY, USA

**Keywords:** Blended learning, Flipped classroom, Graduate education in public health, Online education, Student learning

## Abstract

**Background:**

Blended learning approaches, in which in-person and online course components are combined in a single course, are rapidly increasing in health sciences education. Evidence for the relative effectiveness of blended learning versus more traditional course approaches is mixed.

**Method:**

The impact of a blended learning approach on student learning in a graduate-level public health course was examined using a quasi-experimental, non-equivalent control group design. Exam scores and course point total data from a baseline, “traditional” approach semester (n = 28) was compared to that from a semester utilizing a blended learning approach (n = 38). In addition, student evaluations of the blended learning approach were evaluated.

**Results:**

There was a statistically significant increase in student performance under the blended learning approach (final course point total *d* = 0.57; a medium effect size), even after accounting for previous academic performance. Moreover, student evaluations of the blended approach were very positive and the majority of students (83%) preferred the blended learning approach.

**Conclusions:**

Blended learning approaches may be an effective means of optimizing student learning and improving student performance in health sciences courses.

## Background

Over the past 15 years, an increasing number of courses in the health sciences, as well as courses across colleges and universities, have incorporated online course components. These range from fully online courses to courses that are primary face-to-face with very minor online elements. Of particular interest are courses that adopt a blended learning design, where some course elements are conducted in a traditional classroom setting while other course elements are delivered online [1]. Blended learning involves a combination of online and face-to-face course components, with the notion being that the elements work together as a single, integrated course [2,3]. Sometimes these design decisions are driven by economic, logistical, or other planning considerations [2], whereas other times the decision is made based on relative strengths and weaknesses of different modalities for presenting course information [2,3].

Although the rationale for providing blended learning experiences may vary widely across colleges and universities, from a teaching and learning perspective a critical question is whether such designs are effective at delivering course content and, given the shift from more strongly classroom-based delivery formats, whether blended learning approaches differ from more traditional classroom delivery formats in terms of the learning outcomes students achieve as a result of the course. In addition, it is also important to examine how students experience the blended learning course and their feedback on its effectiveness.

In this paper we examine these questions in the context of a masters-level public health survey course. In a quasi-experimental, non-equivalent control group study, learning outcomes for a blended learning course delivery were compared to those for a more traditional, classroom-based format.

While there is a relatively large literature on effectiveness of fully online course delivery, fewer studies have examined the blended learning approach. This is particularly true for graduate health sciences courses, as much of the literature has focused on undergraduate education. Rhetorical arguments for blended learning have focused on the fact that different learning tasks are naturally suited to particular delivery modalities, with a blending of modalities allowing for a “match” between learning task and delivery mode [3]. Further, arguments have been made that “freeing up” in person class time by moving didactic, lecture presentation online allows for greater engagement in active learning [4].

Whereas there are strong pedagogical arguments in favor of a blended learning approach, the empirical literature on relative effectiveness of blended versus traditional learning approaches is mixed. While some studies have concluded that a blended learning approach is more effective [5], many others have found no differences in outcomes across the two modes of delivery [6-9]. Some work has suggested that the relative efficacy of different modalities may depend on the level of learning outcomes, with online and in person delivery methods being equivalent for lower level skills but in person preferable for higher level skills [10].

Given the mixed findings on the relative merits of a blended learning versus a traditional format for student learning, this paper addresses the question of whether and how, holding course content and learning objectives constant, the shift to blended learning impacts student outcomes. On the one hand, the additional in-person, active learning time afforded by the freeing of class time due to online delivery of lecture components might argue for increased learning in the blended format. On the other hand, shifting lecture material from in person to online potentially downgrades active engagement in learning of those course components given the findings of [10], thus arguing for greater learning in the traditional course format.

This study utilizes a quasi-experimental, non-equivalent control group design to examine the effects of transitioning from a more “traditional” classroom model to a blended classroom model on student learning outcomes. The outcomes of this shift in course delivery were evaluated by three metrics: 1) exam performance; 2) overall course performance; and 3) student course evaluation ratings and open-ended comments.

## Methods

The research reported here was reviewed by the University at Buffalo Social and Behavioral Sciences Institutional Review Board (protocol 426637–1).

### Participants

Participants were 66 graduate students enrolled in either of two semesters of a masters-level course on the social and behavioral sciences in public health (38 students in the blended learning semester, 28 students in the “traditional” comparison semester). Of the 66, 54 were enrolled in the university’s Masters of Public Health program (for which the course is a required part of the core curriculum), 5 were enrolled in a Preventive Medicine residency (for which the course is required), and 7 were enrolled in another university graduate program (for which the course is not required).

### Course description

The course is a master’s level survey course covering the role of the social and behavioral sciences in public health. The course is a required core course for all students enrolled in the university’s Masters of Public Health program and for medical school graduates completing the university’s residency program in preventive medicine. In addition, the course attracts a small number of PhD students from a range of disciplines including nursing, social work, communications, and psychology.

During the “baseline” semester (Fall 2011), the course was taught in a relatively traditional format. Students completed out of class reading assignments each week (typically 2–4 journal articles or book chapters), but all presentation of non-reading course content was done in class through instructor lecturing. Class time also included active learning activities, including small group work and class discussions. Approximately 60% of in class time was lecture-based, with the remaining 40% involving active learning.

During the “blended learning” semester (Fall 2012), all pre-planned didactic content presentation was pre-recorded and posted online for student viewing prior to the week’s in class sessions. Class time was then almost entirely (at least 80% of class time) devoted to active learning approaches. In class lecturing only took place when necessary to clarify points of student confusion or where integrating lecture presentation with an active learning activity was necessary for the activity’s successful implementation.

Notably, the learning goals and the course content remained the same during the two semesters. Course readings were nearly identical; where readings changed from the baseline to the blended semester, it was in situations where information presented in a reading and information presented in the recorded lecture overlapped. The key changes from baseline to blended, then, were: a) presentation of didactic lecture components online rather than in class; and b) given the shift to online lecture presentation, freeing of in class time for more in depth, active learning engagement with the course concepts.

### Evaluation components

#### Exams

The course was broken up into three units, each approximately 3 1/2 weeks long. Following each unit, students completed a non-cumulative unit exam. Each unit exam consisted of 10 multiple choice and 4 short answer questions. Exams were purposefully held constant across the two semesters to allow for performance comparisons. The only differences were changes to 1–2 multiple choice questions on Exams 1 and 2 to correct problematic items.

#### Overall course point total

In addition to exams, the overall course point total was based on performance on writing assignments, a capstone end of semester project, and participation in in-class and out-of-class activities.

#### Student evaluation ratings and open-ended comments

At the end of the semester, student anonymously completed a standardized, school-wide course evaluation. The evaluation included closed-ended ratings of both the quality of the course and the quality of the instructor. For both of these ratings, students responded on a 5 points scale with endpoints of 1 = unacceptable and 5 = one of the best. Students then responded to two open-ended questions: “Please comment on elements of the course you found particularly effective” and “Please comment on course improvements you would suggest.” In addition, a supplemental evaluation question was added in which students were asked “Given the option, would you prefer to take the course in the blended format we used this semester or in a more “traditional” lecture in class format?”

### Analysis plan

Prior to analysis, four students were removed from the dataset. Two students (one from each semester) were removed because they dropped the class shortly after the first exam. In addition, two students took the course in the baseline semester and then re-took the course in the blended learning semester due to failure to receive a grade of B or higher in the course (required for graduate credit). These students were removed from the blended learning semester data as that was their second time taking the course. One of two students failed the baseline semester course due to academic dishonesty on a course project. For that student, exam scores were included in the dataset but the final course point total was not (given that the course total reflected the grading consequences of academic dishonesty rather than course performance).

Because undergraduate GPA was not available for all students, we tested whether students for whom GPA data was available differed from those for whom it was not available. There were no differences in any course grading component based on availability of GPA. Moreover, there were no GPA availability x semester interactions on any outcome variable; all *F-*tests *ns.*

The key test for the relative efficacy of the blended learning approach is comparison of exam performance and course point totals across the two semesters. Given the non-equivalent control group design, a primary threat to validity of this test is differences in student characteristics across the two semesters. For that reason, prior to analyses we examined equivalence of the students on prior academic performance (indexed by undergraduate GPA), program of study, and gender. There were no differences across the two semesters (see *Participants* above). Although this lack of differences strengthens confidence in the approach, to further address the possibility of lack of comparability we conducted all analyses controlling for undergraduate GPA. The exam analyses were conducted using repeated measures ANCOVA with the three exam scores as a repeated measures outcome variable, semester as a categorical predictor variable, and undergraduate GPA as a continuous covariate. Final course point total was similarly modeled using univariable ANCOVA with the final course point total as the continuous outcome variable and the above predictors and covariates modeled.

Importantly, all outcomes analyses were run three ways: 1) without controlling for undergraduate GPA, 2) controlling for undergraduate GPA and including in analysis only those students for whom GPA was available, and 3) using mean score substitution (by semester) to estimate GPA for those students for whom it was unavailable and then controlling for undergraduate GPA for all students. The pattern of mean differences (i.e., which semester had a higher or lower score) was the same across all three methods. Given this, in all data reported here, means and standard deviations are unadjusted for GPA (such that descriptive data is based on the full dataset); reported significance tests reflect differences controlling for GPA for students for whom a specific undergraduate GPA was available.

Finally, to examine student feedback on the blended learning approach, open-ended course evaluation feedback from the blended learning semester was content analyzed by the author. Responses were coded into a series of feedback categories (see Table 1) constructed after an initial read-through of the feedback and refined during the process of coding. Coding was non-exclusive (i.e., a particular student comment could be coded as belonging to more than one outcome category). In addition, scores on two closed-ended evaluation items were compared across semesters using linear regression with evaluation score as a continuous outcome measure and semester as a categorical predictor variable. Because student evaluations are anonymous and separate from other course components, it was not possible to include the covariates in this analysis.

**Table 1:** Student evaluations, open-ended feedback for blended learning semester

**Response category**	**Percentage of responses**
**Effective course components**	
Online lecture-ette segments	54%
	Example: “I found the video lecturettes to be extremely effective.”
In class activities/discussions	44%
Example: “Class discussions/activities to provide real-world context of the theories/ideas”	
Online learning support materials (reading questions, road maps)	16%
Example: “I feel the weekly road maps and the questions posed prior to the readings and lecture-ettes were particularly helpful and served as a form of study guide.”	
Weekly online learning journals	10%
Example: “Weekly learning journals were a good way to make us go over what we learned that week, making it easier to remember.”	
**Suggested course improvements**	
Learning journals	18%
Example: “The learning journals got tedious as things in the semester began to pick up.”	
Readings (most comments about length, amount, “dryness”, difficulty)	15%
Example: “ Some of the readings required for class were dry and hard to pull out the key concepts without going over them in class.”	
Balance of in class activity to in class lecture time	15%
Example: “Blended course structure that included somewhat more brief in-class lecture would be helpful, at least for my personal learning style.”	
Group project (most comments about how to structure/grade) Example: “I would suggest no group project. Group projects are the worst and take way more time to complete than individual projects.”	15%

Note: content categories in table represent any categories included by more than 10% (n ≥ 4) of students.

## Results

Prior to examining effects of student outcomes, characteristics of the participants during the two semesters were compared. Undergraduate GPA data was available for 42 of the 66 students; GPA did not differ significantly by semester; baseline M = 3.56 (SD = 0.51), blended M = 3.49 (SD = 0.24), *F*(1,40) < 1, *ns*. In addition to GPA, students in the two semesters did not differ in terms of gender composition (*χ*^2^(1) = 0.14, *ns)* or program of studies (*χ*^2^ (2) = 1.42, *ns*).

### Exam scores

Scores for the three unit exam by semester are reported in Table 2. Overall, exam performance was higher in the blended learning semester relative to the baseline semester, repeated measures semester effect *F*(1,39) = 6.12, *p* < .05, partial η^2^ = 0.14. Followup testing for individual exams revealed that, as can be seen in the table, exam scores for the blended learning semester were statistically significantly higher than for the baseline semester for both Exams 1 and 2. For exam 3, there was not a significant difference across the two semesters. Effect sizes for the differences in performance across the exams are also reported in Table 2.

**Table 2:** Exam scores and final course scores by semester

**Course score component (total possible points)**	**Baseline, traditional delivery M (SD)**	**Blended learning semester M (SD)**	**Effect size for semester differenced**	**ANCOVA semester effect, controlling for undergraduate GPA *F *** **(39 *df), *** **partial η**^**2**^
Exam 1 (15 points)	**13.25 (1.35)**	**13.61 (0.69)**	**0.35**	**5.97, *p*** **< .05, partial** η^**2**^ **= 0.13**
Exam 2 (15 points)	**13.61 (0.99)**	**14.10 (0.89)**	**0.51**	**14.24, *p*** **< .001 partial** η^**2**^ **= −0.27**
Exam 3 (15 points)	13.76 (0.96)	13.54 (0.53)	−0.29	0.90, *p* = 0.35 partial η^2^ = 0.023
Total course point Total (100 points)	**91.76 (4.95)**	**93.92 (2.45)**	**0.57**	**12.12, *p*** **< .001, partial** η^**2**^ **= 0.24**

In the data table, means and standard deviations are unadjusted for GPA; significance tests reflect differences controlling for GPA for students for whom a specific undergraduate GPA was available.

### Overall course score/point total

Table 2 also presents overall course scores/point totals for the two semesters. The overall course total was significantly higher during the blended learning semester relative to during the baseline semester, Cohen’s *d* = 0.57, a medium effect [11].

### Student evaluations – closed-ended ratings

Ratings from student evaluations of the course are presented in Table 3. As can be seen I in the table, there were no differences in numerical ratings of either the course or the instructor across the two semesters; for both questions, *t*(66) < 1, *ns.*

**Table 3:** Student evaluations, comparison by semester

	**Fall 2011 (N = 28)**	**Fall 2012 (N = 38)**
**1) What is your overall rating of this course?**	3.8	3.9
**2) What is your overall rating of the instructor’s teaching effectiveness?**	4.1	4.1

### Student evaluations – open-ended feedback

Student responses to the open-ended course evaluation questions are summarized in Table 1. The open-ended questions on “Please comment on elements of the course you found particularly effective” and “please comment on course improvements you would suggest” were content analyzed. Of particular note, 27 students (73%) mentioned an aspect of the blended learning experience (online lecture-ette segment and/or in-class activities) as an effective element of the course. By contrast, only 5 students (15%) commented about problems with the relative balance of online and in-class components.

### Student evaluations – preference for blended versus traditional format

In addition to the standard course evaluation questions, students were asked to indicate their preference for the blended learning approach relative to a more “traditional” course approach. Of the students in the course, the blended approach was preferred by 83% (n = 33), whereas only 10% (n = 4) preferred the traditional approach. The remaining 8% (n = 3) wrote in a response indicating a preference for a “combination” of the two approaches.

## Discussion

This examination of the relative effectiveness of using a blended versus a “traditional” approach to delivering course content in a masters-level public health course revealed several interesting aspects of effectiveness. First, student outcomes during the blended learning semester were by and large higher than during the traditional learning semester – both exam performance and overall course performance was higher under the blended learning approach relative to the traditional classroom delivery approach. Using Cohen’s [12] guidelines for describing effect sizes, the final course performance difference was a medium effect. This was true even after accounting for pre-existing student differences in academic achievement.

Second, student feedback concerning the blended learning approach was predominantly positive. There were substantially more positive feedback comments concerning the blended learning approach than there were negative comments about the blended learning techniques, and the negative feedback that was received predominantly concerned details of how the blended learning components were implemented rather than negativity about the approach *per se.* Finally, the students reported an overwhelming preference for the blended learning approach as opposed to a more traditional delivery method.

### Implications and future directions

What might account for the effects of the blended learning approach on student learning? Arguably, one reason for these effects might be that, given the shift to blended learning, there was additional “time on task” – students spent more time grappling with each course concept than before, and the integration of active learning activities for in-class time allowed for structuring “higher order” engagement with the concepts (analyzing them, applying them to address novel situations, etc.). Time on task is one of the best practices for university education [13]. Appropriate use of technology can increase time on task and thus improve learning [14].

A second possibility is raised from informal comments from students. Several students commented that they liked the online presentation of lecture material because it allowed them to pause when they noticed their attention flagging, rewind when there was a point they wanted to hear again, and revisit after a class session if they wanted to clarify a muddy point. Thus, the blended learning approach might also alter how students engage with lecture material in ways that further optimize learning.

There are a number of fruitful directions for additional scholarship on this topic. First, given that blended learning is defined as any mixing of in person and online course components, it seems reasonable to ask what the optimal degree of blending would be for a particular type of course, type of student, or type of program. What is the optimal mix of in person versus online components? Within that, what is the optimal mix of didactic presentation versus active engagement activities? Better specifying how different “blends” of course components influence learning would allow more effective implementations of blended learning approaches. It might also have the effect of explaining the mixed findings for the relative effectiveness of blended learning approaches (discussed in the *Introduction*). Perhaps the relative effectiveness of blended versus more traditional course approaches is dependent on how blended the course is and the nature of the blended materials.

Second, given that blended learning offers a mixture of pedagogical techniques, delivery mechanisms, and student engagement strategies, it would be valuable to examine which specific components of a blended learning approach contribute the most to student success. To date most examinations of the effectiveness of blended learning have examined the course as a whole. A more fine grained examination of which course components contribute the most to the effectiveness (or lack thereof) of a blended learning approach would both advance our understanding of how and why blended approaches impact student learning but would also provide more detailed guidance for implementing optimally effective blended learning courses.

### Limitations

There are, of course, limitations to the work presented here that should be acknowledged when considering the findings and their implications. First, the study design was not truly experimental in that students were not randomly assigned to semester and, thus, to the blended versus traditional learning approach. While steps were taken to address this potential threat to internal validity (e.g., testing for equivalence on a number of demographic characteristics, controlling for prior academic achievement in analyses), it is important to acknowledge that other factors might account for at least a portion of the difference in learning outcomes across semesters.

Second, the outcome measures assessed student learning via course evaluation performance, but did not include assessment of the processes through which those differential learning outcomes occurred. Future research would be beneficial to examine, for example, whether the blended learning approach led to greater student engagement with readings and lecture materials, more time studying, differential learning strategies, or other possible process explanations for the difference in learning outcomes.

Third, it is important to note that the instructor was fully aware of and engaged in the change from the “traditional” learning approach to the blended course approach. This raises the possibility that unmeasured differences in instructional approach, instructor enthusiasm, or other factors might contribute to the differences in findings reported here. Although the consistency in student ratings of the instructor over the two semesters does somewhat address this concern, the possibility of unmeasured instructor effects cannot be ruled out. In addition, the minor changes to examinations (described in *Methods* above) might have had some impact on performance, although even the largest possible effect that these exam change could have on performance would not account for the differences in results across the two semesters.

Finally, in terms of estimating the potential magnitude of the effect of the blended learning approach, it should be acknowledged that the comparison semester, although structured as a “traditional” learning course rather than a blended course, still involved a fair amount of active learning approaches in the classroom. Thus, the data presented here might underestimate the potential benefit of shifting from a truly “traditional” approach (i.e., one in which the vast majority of classroom time is used for faculty lecturing) to a blended course format.

## Conclusions

Shifting presentation of course content from a traditional approach to a blended learning approach, while keeping the intellectual content and course evaluation consistent, lead to an increase in student learning as assessed by exam performance and overall course point totals. Moreover, student feedback about the approach was very positive and students overwhelmingly preferred the blended approach to a more traditional course structure. Well implemented blended learning approaches may have strong potential for improving student learning outcomes in health sciences courses.

### Ethical approval

Ethical approval was waived (secondary data analysis of deidentified data) by the University at Buffalo Social and Behavioral Sciences Institutional Review Board, January 2013.

## Competing interests

The author declares that he has no competing interests.

## Authors’ contributions

MTK had responsibility for all aspects of this paper.

## Pre-publication history

The pre-publication history for this paper can be accessed here:

http://www.biomedcentral.com/1472-6920/14/47/prepub
